# Determinants of catastrophic health expenditures in Iran: a systematic review and meta-analysis

**DOI:** 10.1186/s12962-020-00212-0

**Published:** 2020-05-15

**Authors:** Leila Doshmangir, Mahmood Yousefi, Edris Hasanpoor, Behzad Eshtiagh, Hassan Haghparast-Bidgoli

**Affiliations:** 1 Tabriz Health Services Management Research Center, Iranian Center of Excellence in Health Management, Tabriz University of Medical Sciences, Tabriz, Iran; 2 Social Determinants of Health Research Center, Health Management and Safety Promotion Research Institute, Tabriz University of Medical Sciences, Tabriz, Iran; 3 Department of Health Policy and Management, School of Management and Medical Informatics, Tabriz University of Medical Sciences, Tabriz, Iran; 4 Department of Health Economics, School of Health Management and Medical Informatics, Tabriz University of Medical Sciences, Tabriz, Iran; 5 Research Center for Evidence-Based Health Management, Maragheh University of Medical Sciences, Maragheh, Iran; 6 Institute for Global Health, University College London, London, UK

**Keywords:** Catastrophic health expenditures, Health equity, Fair health financing, Iran

## Abstract

**Background:**

Catastrophic health expenditures (CHE) are of concern to policy makers and can prevent individuals accessing effective health care services. The exposure of households to CHE is one of the indices used to evaluate and address the level of financial risk protection in health systems, which is a key priority in the global health policy agenda and an indicator of progress toward the UN Sustainable Development Goal for Universal Health Coverage. This study aims to assess the CHE at population and disease levels and its influencing factors in Iran.

**Methods:**

This study is a systematic review and meta-analysis. The following keywords and their Persian equivalents were used for the review: Catastrophic Health Expenditures; Health Equity; Health System Equity; Financial Contribution; Health Expenditures; Financial Protection; Financial Catastrophe; and Health Financing Equity. These keywords were searched with no time limit until October 2019 in PubMed, Web of Science, Scopus, ProQuest, ScienceDirect, Embase, and the national databases of Iran. Studies that met a set of inclusion criteria formed part of the meta-analysis and results were analyzed using a random-effects model.

**Results:**

The review identified 53 relevant studies, of which 40 are conducted at the population level and 13 are disease specific. At the population level, the rate of CHE is 4.7% (95% CI 4.1% to 5.3%, n = 52). Across diseases, the percentage of CHE is 25.3% (95% CI 11.7% to 46.5%, n = 13), among cancer patients, while people undergoing dialysis face the highest percentage of CHE (54.5%). The most important factors influencing the rate of CHE in these studies are health insurance status, having a household member aged 60–65 years or older, gender of the head of household, and the use of inpatient and outpatient services.

**Conclusion:**

The results suggest that catastrophic health spending in Iran has increased from 2001 to 2015 and has reached its highest levels in the last 5 years. It is therefore imperative to review and develop fair health financing policies to protect people against financial hardship. This review and meta-analysis provides evidence to help inform effective health financing strategies and policies to prioritise high-burden disease groups and address the determinants of CHE.

## Background

Healthcare is a natural right of every human being that is necessary in all the stages of life and must not be affected by their wealth or income [1, 2]. Presently, the rising costs of healthcare services and their impact on the economy have become major concerns for health policy makers [3–6]. Health systems are therefore seeking financing mechanisms that will improve access to quality health services in underserved communities [7, 8]. The reliance on out-of-pocket expenditure to finance health services is a common feature in many low- and middle-income countries. Households without adequate financial protection face the risk of incurring large unanticipated medical expenditures. These unforeseen expenditures may lead to indebtedness, a reduction in living standards, and ultimately impoverishment [9, 10].

Improving financial protection to minimize the extent to which households incur catastrophic health expenditures (CHE) and are pushed into poverty due to high medical spending has received substantial attention. The link between poverty and health is well established, and in 2015 CHE was included as a key indicator to monitor progress toward the UN Sustainable Development Goal (SDG) for Universal Health Coverage. More recently, health insurance has been put forward as an instrument to provide financial protection and to achieve universal coverage [1, 3, 7]. As a result, the World Health Organization (WHO) has underlined the importance of protections against CHE and considers fair financing to be a key objective for health systems. Fair health financing ensures that households do not pay beyond a certain proportion of their total income for health out-of-pocket payments (OOPs) and protects them against impoverishment due to CHE [10].

CHE can occur in all countries at all stages of development. The CHE rate is one of the main factors used to calculate fairness in health financing [11, 12]. Health expenditures are considered catastrophic when they exceed a certain amount (e.g. 10%) in relation to the household’s income, expenditure, or the ability to pay [12, 13]. CHE can either be a proportion of total income/consumption (e.g. 10%) or the ability to pay. Ability to pay is defined as the capability to use money for health expenditure with respect to annual household income that is not required for subsistence, for example household income less spending on food or housing. Health expenditure not exceeding 5% of annual household income is a common benchmark of ability to pay [14]. This is because there is starting to be a movement away from ability-to-pay (i.e. non-food expenditure as a denominator). For example, the 10% threshold is used for the UN SDGs indicator and for UHC progress tracking by the World Bank and WHO [15].

CHE can lead to a reduction in consumption in the short-term and the use of savings, sale of assets, and borrowing in the long-term, thus reducing the household’s living standards [16]. Globally, more than 150 million people are exposed to CHE annually, and around 100 million are pushed into poverty because of OOPs [17]. Various studies have been conducted on CHE in Iran at the population level and across diseases, and rates of CHE ranging between 2.5 and 72.5% have been reported [17–19].

The purpose of the present research was to systematically review the studies investigating CHE in Iran and to synthesize their results across populations, diseases, and vulnerable groups, thus providing new insights into CHE in Iran as an indicator of fair health financing.

## Methodology

This study is a systematic review and meta-analysis of the studies carried out on CHE in Iran based on the Preferred Reporting Items for Systematic Reviews and Meta-Analyses (PRISMA) guidelines [20]. All the different phases of the review, from the search to quality assessment of the studies, were independently performed by two reviewers and disagreements were examined by a third reviewer. Studies were accessed from a number of Persian and English language databases, including PubMed, Web of Science, Scopus, ProQuest, ScienceDirect, Embase, MagIran, IranMedex, SID, and IranDoc as well as Google Scholar. In addition, the bibliographies of selected studies were searched to identify additional studies. All studies conducted up to October 2019 were included. The following keywords and their Persian equivalents were used to search the databases: Catastrophic Health Expenditures; Health Equity; Health System Equity; Financial Contribution; Health Expenditures; Financial Protection; Financial Catastrophe; and Health Financing Equity. The operators “And” and “Or” were also used to broaden the search. A detailed search strategy is included in Additional file 1.

### Inclusion criteria

#### Types of studies

The inclusion criteria were: (1) any primary study in English or Persian measuring and reporting catastrophic health expenditures, and/or factors affecting them across demographics and diseases, and (2) studies conducted in Iran.

#### Types of participants

The participants are households or patients who lived in Iran.

#### Types of intervention

Factors that influence the catastrophic health expenditure of households.

#### Types of outcomes

Catastrophic health expenditure: Payment is considered catastrophic when a household has to cut its basic living expenses over 1 year in order to afford the medical expenses of its household member(s).

### Exclusion criteria

Methodological studies and studies that do not measure or report CHE and using approches other than CHE to measure equity in health financing were excluded.

### Quality assessment

To assess the quality of the studies, first the name of the journals and authors were concealed. The studies were then given to two members of the research team to independently examine the inclusion and exclusion criteria, with a third researcher resolving the disagreements. As the majority of the studies included in this review are observational, the STrengthening the Reporting of OBservational studies in Epidemiology (STROBE) checklist [21] was used in quality assessment. This checklist consists of five main domains (title and abstract, introduction, results, discussion and other information) and 22 sections, with a minimum score of 0 and a maximum score of 44. Checklist items were rated on a three-point scale (yes = 2, cannot tell = 1, and no = 0). Studies were divided into three groups: (1) high quality (a score higher than 30) (2) moderate quality (a score between 16 and 30), and (3) low quality (a score less than 16). Studies with quality scores higher than 16 were included in the meta-analysis stage.

### Data extraction

The general characteristics of the studies were extracted and presented in a data extraction form. This form included first author’s name, year of publication, study design, data collection period, location/region, sample size, data collection method, and catastrophic health spending rate as well as factors affecting it.

### Statistical analysis

Study heterogeneity was investigated using Cochran’s Q and $I^{2}$ index. An I^2^ > 50% or a P-value for the Q test < 0.10 indicates significant heterogeneity [22]. Since the results of Q test and $I^{2}$ index indicated significant heterogeneity between the studies, a random effects model was used for meta-analysis and synthesized results were obtained from the Comprehensive Meta-Analysis (CMA) software, version 3. Factors affecting the rate of CHE were extracted and classified by population and disease. The possibility of publication bias was assessed using visual inspection of a funnel plot.

## Results

**Fig. 1:**
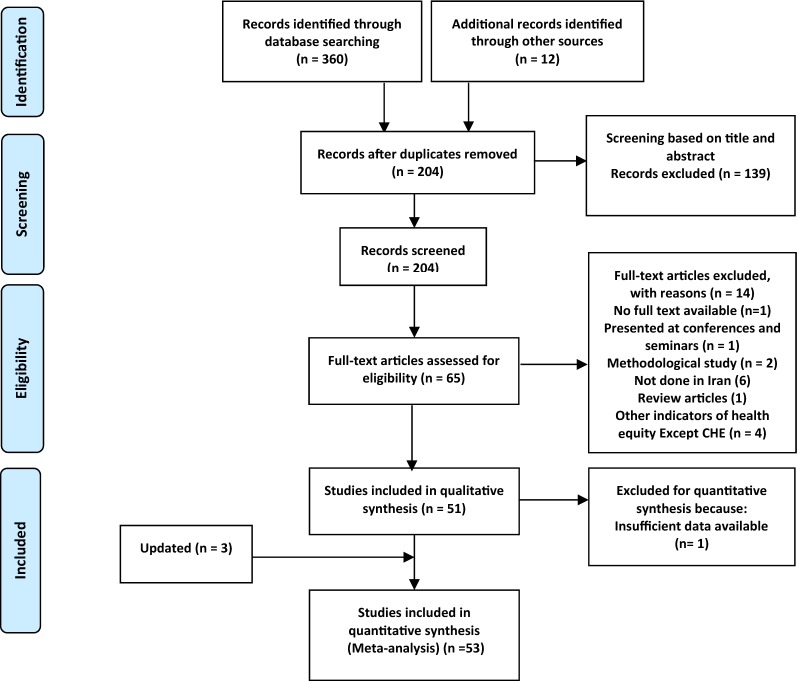
Flow diagram of studies included in the meta-analysis

**Fig. 2:**
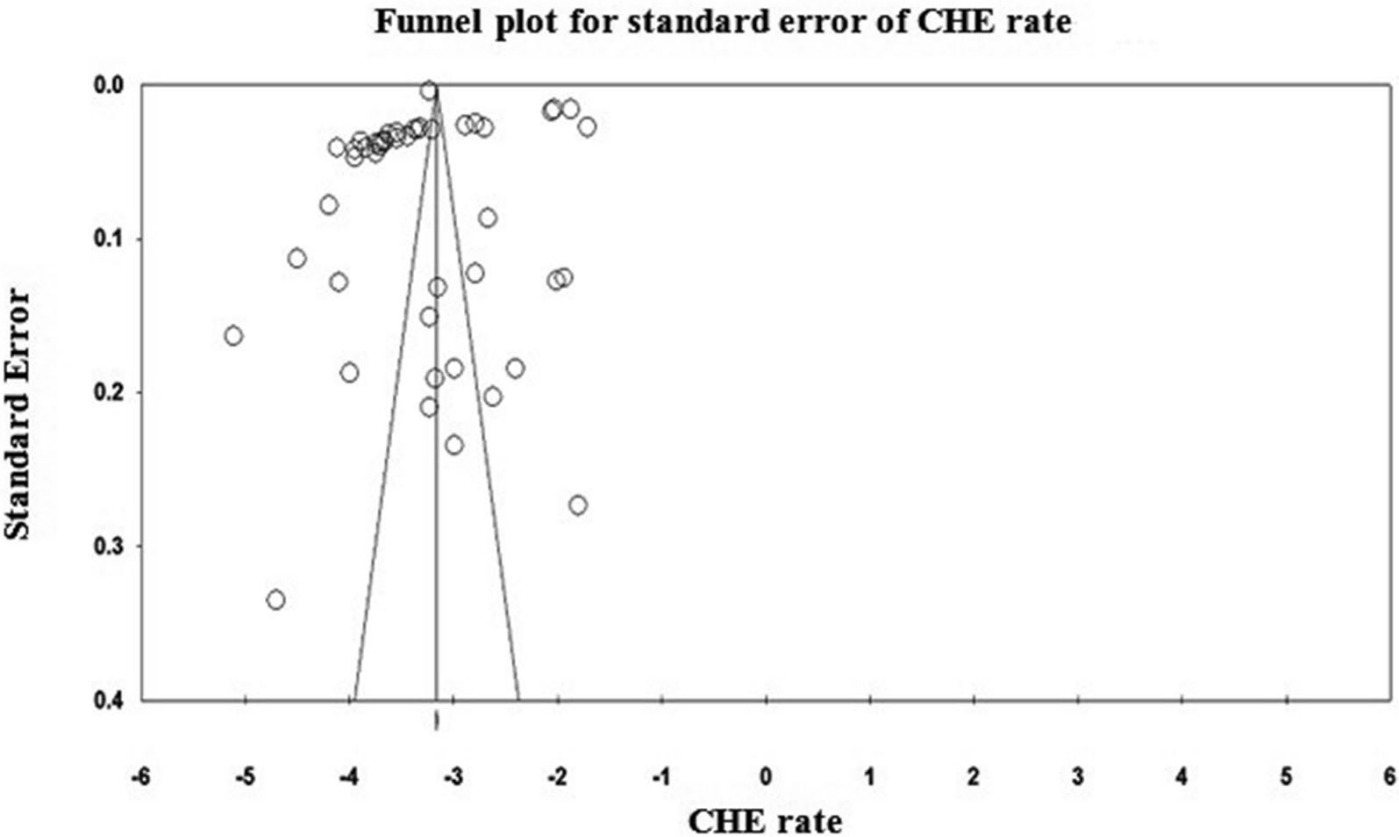
Funnel plot for evaluation of publication bias

**Fig. 3:**
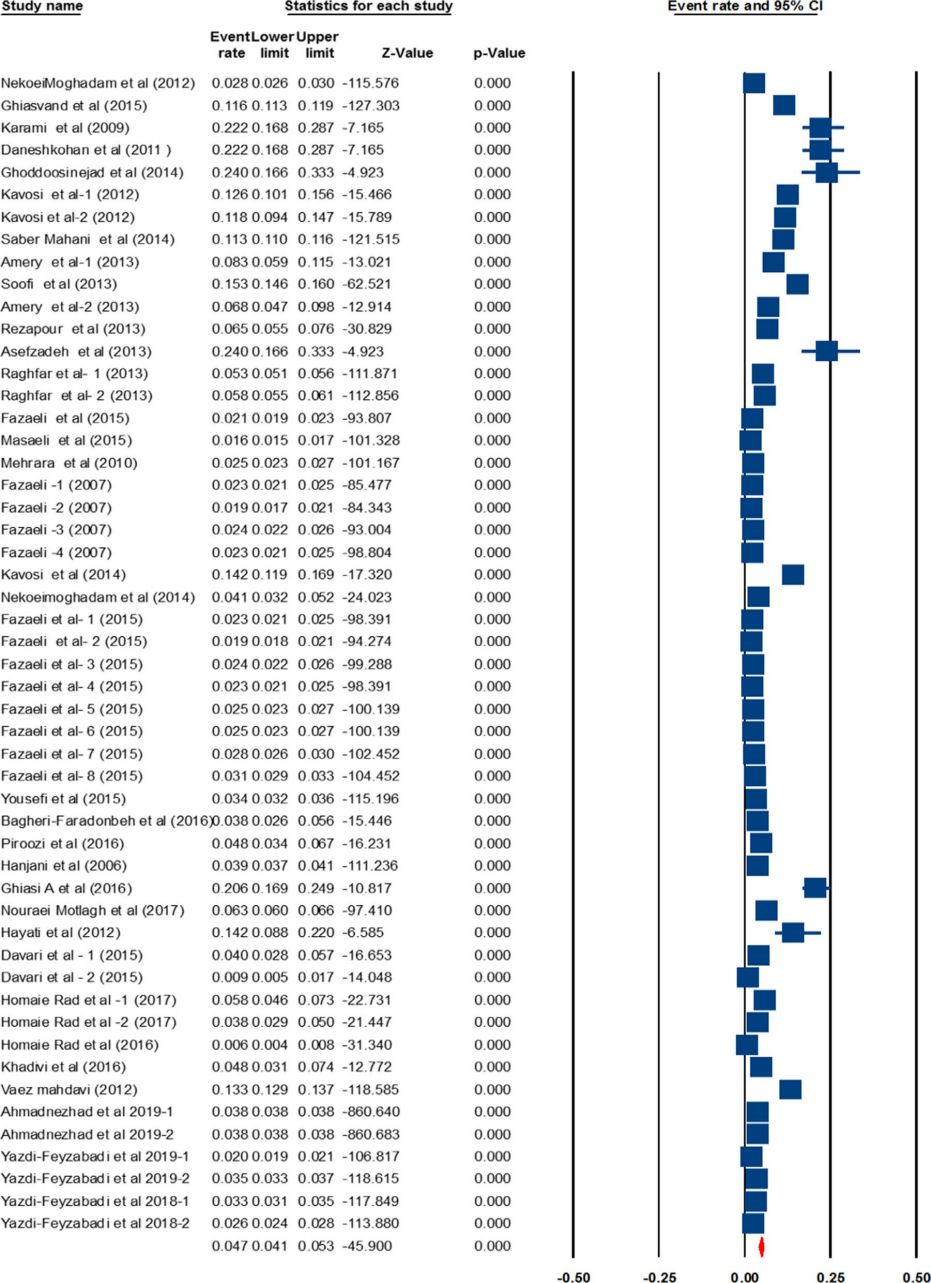
The pooled estimate of CHE prevalence in Iran (population level)

A systematic search of the keywords identified 360 studies in the selected databases. An additional 12 studies were also obtained though manual searches of the bibliographies of the final studies (Fig. 1). In total, 52 papers [1, 3, 11, 12, 18, 19, 23–63] were included in the meta-analysis stage (Figs. 1, 2). These studies were classified into two groups, based on whether they investigate CHE across demographics (40 studies) or diseases (13 studies). The general characteristics of the studies and the data extracted from them are provided in Tables 1 and 2. Analysis of publication bias revealed that no publication bias was identified by Egger’s line regression test (P > 0.05). A visual inspection of the symmetry graphic in the funnel plot indicated no evidence of publication bias or small-study effects (Fig. 3).

**Table 1:** The data extraction and quality of the studies (population level)

Study quality	CHE (%)	Data collection method	Sample size	Population	Years of study	Publication type—language	Study design	Author (year)	N
1	Nekoei-Moghadam et al. (2012)	Descriptive–analytical study	Article—English	2008	Iranian households	39,008	Secondary data	2.8%	Good
Determinants of exposure to CHE: use of outpatient service, drug addiction cessation services, Inpatient service—household size (3 ≤ x < 6) (+)—economic status—pharmaceutical expenses—health insurance									
2	Ghiasvand et al. (2015)	Descriptive study	Article—English	2013–2014	Iranian households	Total: 38,318 19,437 (rural) 18,888(urban)	Secondary data	Rural: 11.7% Urban: 11.45%	Good
Determinants of exposure to CHE									
3	Karami et al. (2009)	Descriptive study	Article—English	2008	Kermanshah	189	questionnaire	22.2%	Medium
Determinants of exposure to CHE									
4	Daneshkohan et al. (2011)	Descriptive study	Article—English	2008	Kermanshah	189	Questionnaire	22.2%	Good
Determinants of exposure to CHE									
5	Ghoddoosinejad et al. (2014)	Cross-sectional descriptive study	Article—English	2013	Ferdows	100	Questionnaire	24%	Medium
Determinants of exposure to CHE: use of dentistry services									
6	Kavosi et al. (2012)	Longitudinal study	Article—English	2003 and 2008	South-west Tehran	579 (2003) 592 (2008)	Questionnaire	12.6% (2003), 11.8% (2008)	Good
Determinants of exposure to CHE: economic status—member over 65 years (+)—disabled members—health insurance- use of dentistry services, outpatient service, inpatient service									
7	Saber-Mahani et al. (2014)	Cross-sectional study	Article—Persian	2011	Tehran	34,700	Secondary data	11.3%	Medium
Determinants of exposure to CHE: number of members under 5 years (+)—number of members over 65 years (+)—employed head—education status of household head (−)—chronic disease members—health insurance—age of household head (+)—equivalent household size (−)—income deciles (+)—per capita household expenditure (−)—number of the employed persons in household									
8	Amery et al. (2013)	Cross-sectional study	Article—Persian	2011	Yazd	386	Questionnaire	8.3%	Medium
Determinants of exposure to CHE: use of inpatient services—household size (> 7) (+)—members under 5 years (−)—use of medical services and diagnosis									
9	Soofi et al. (2013)	Cross-sectional study	Article—Persian	2001	Iranian households	10,300	Secondary data	15.31%	Medium
Determinants of exposure to CHE: living in the urban (−)—household size (+)—member with chronic illness—member in need of care—economic status—health insurance—use of outpatient service									
10	Kavosi et al. (2009)	Longitudinal study	Article—Persian	2003–2008	Tehran	579 (2003), 592 (2008)	Questionnaire	12.6% (2003), 11.8% (2008)	Medium
Determinants of exposure to CHE: use of inpatient service, dentistry services—member over 65 years (+)—member in need of care—number of use of outpatient services—economic status									
11	Amery et al. (2012)	Cross-sectional study	Article—Persian	2012	Mashhad	384	Questionnaire	6.77%	Medium
Determinants of exposure to CHE: household size (> 7) (+)—health insurance—use of inpatient service, dentistry services, medicinal and diagnostic services—member over 65 years (+)—pharmaceutical expenses—members under 5 years (−)									
12	Rezapour et al. (2013)	Cross-sectional study	Article—English	2013	Tehran	2200	Interviews, and Questionnaire	6.45%	Good
Determinants of exposure to CHE: number of use of outpatient services—education status of household head (+)—household size (+)—preschool children living in household (−)—member with chronic illness									
13	Aeenparast et al. (2016)	Review literature on studies	Article—Persian	Not reported	Iranian households	19 papers	–	2.5% to 72.5%	Weak
Determinants of exposure to CHE									
14	Asefzadeh et al. (2013)	Cross-sectional–descriptive–analytical study	Article—Persian	2011	Qazvin	100	Questionnaire	24%	Medium
Determinants of exposure to CHE: use of dentistry servicesDeterminants of exposure to CHE: use of dentistry services									
15	Raghfar et al. (2013)	Longitudinal study	Article—Persian	1984 to 2010	Iranian households	30,000 households in each year	Secondary data	6.78% to 5.76% (rural) 3.9% to 5.76% (urban)	Weak
Determinants of exposure to CHE									
16	Fazaeli et al. (2015)	Cross-sectional–descriptive–analytical study	Article—English	2010	Iranian households	28,997	Secondary data	2.1%	Medium
Determinants of exposure to CHE: living in the urban (−)—number of members over 65 years (+)—education status of household head (+)—employment situation of household head—number of the employed persons in household—expenditure deciles (+)—equivalent household size (+)									
17	Masaeli et al. (2015)	Descriptive–analytical study	Article—Persian	2011	Iranian households	38,437	Secondary data	1.56%	Medium
Determinants of exposure to CHE									
18	Mehrara et al. (2010)	Longitudinal study–descriptive–analytical study	Article—Persian	2003–2007	Iranian households	31,283 (2007) 2003–2004-2005-2006 (not reported)	Secondary data	2.3% (2003) 1.9% (2004) 2.4% (2005) 2.3% (2006) 2.5% (2007)	weak
Determinants of exposure to CHE: living in the urban (−)—number of members over 60 years (+)—number of members under 12 years (+)—health insurance—employment situation of household head—number of members employed in the household (+)—marital status (single head) (+)—per capita infrastructure residential area of the household, wealth index (−)—equivalent household size (+)—expenditure deciles (+)—equivalent per capita household expenditure (+)									
19	Fazaeli (2007)	Longitudinal study	Thesis—Persian	2003–2006	Iranian households	23,134 (2003) 24,534 (2004) 26,895 (2005) 30,910 (2006)	Secondary data	2.28% (2003) 1.9% (2004) 2.36% (2005) 2.26% (2006)	Medium
Determinants of exposure to CHE: age of household head (−)—number of members employed in the household (−)—health insurance—members over 60 years (+)—living in the urban (−)—education status of household head (−)—employment situation of household head—per capita household expenditure— (+)per capita infrastructure residential area of the household, wealth index (−)									
20	Kavosi et al. (2014)	Cross-sectional study	Article—English	2012	Shiraz	761	Questionnaire	14.2%	Good
Determinants of exposure to CHE: Economic status (−)—use of dentistry services, inpatient services, physician visits—frequency of use of outpatient services—health insurance—supplementary insurance status of household head—member in chronic need of medical care- living in the urban (−)									
21	Nekoei-moghadam et al. (2014)	Descriptive–analytical retrospective	Article—Persian	2008	Kerman	1477	Secondary data	4.1%	Good
Determinants of exposure to CHE: living in the urban (+)—use of inpatient services, outpatient services, dental care services									
22	Fazaeli et al. (2015)	Longitudinal study	Article—English	2003 to 2010	Iranian households	23,134 to 38,170 for each year	Secondary data	2.28% (2003) 1.91% (2004) 2.37% (2005) 2.27% (2006) 2.49% (2007) 2.46% (2008) 2.82% (2009) 3.06% (2010)	Medium
Determinants of exposure to CHE									
23	Yousefi et al. (2015)	Cross sectional–descriptive study	Article—Persian	2011	Iranian households	36,071	Secondary data	3.38%	Medium
Determinants of exposure to CHE									
24	BagheriFaradonbeh et al. (2016)	Cross-sectional study	Article—Persian	2013	Tehran	625	Interview and observation using a Questionnaire	3.8%	Medium
Determinants of exposure to CHE: use of inpatient services- education status of household head (−)—number of use of health services—informal payment (+)—member over 65 years (+)									
25	Piroozi et al. (2016)	Cross-sectional, descriptive–analytical study	Article—English	2015	Sanandaj	646	Face-to-Face Interviews—Questionnaire	4.8%	Good
Determinants of exposure to CHE: supplementary health insurance—gender of the head of household (female)(+)—economic status—members over 65 years(+)—disabled member and in need of care—use of inpatient services, dental care services, rehabilitation services									
26	Hanjani et al. (2006)	Cross-sectional study	Article—Persian	2002	Iranian households	32,152	Secondary data	3.94%	weak
Determinants of exposure to CHE: age of household head (+)- living in the urban (−)—health insurance—education status of household head (−)—employment situation of household head—marital status (married) (+)—gender of the head of household (male) (+)—household size (−)									
27	Ghiasi (2016)	Cross-sectional, descriptive–analytical study	Article—Persian	2013–2014	Zabol	393	Questionnaire	20.6%	Good
Determinants of exposure to CHE: education status of household head (−)—pharmaceutical expenses									
28	Rezapour et al. (2016)	Cross-sectional study	Article—Persian	2013	Tehran	625	Questionnaire	–	Medium
Determinants of exposure to CHE: education status of household head (−)—health insurance—members over 60 years (+)—inpatient service—informal payment (+)—number of use of health services									
29	Fattahi et al. (2015)	Cross-sectional study–case study	Article—Persian	2012–2013	Hossein Abad district of Uremia	300	Questionnaire	–	Medium
Determinants of exposure to CHE: wealth index(−)—gender of household head (male) (−)—household size (+)—members under 12 years (+)—employment situation of household head—number of use of inpatient services—health insurance—supplemental insurance									
30	Nouraei-Motlagh S (2017)	Descriptive-analytical–retrospective study	Article—Persian	2012	Deprived states of Iran	22,057	Secondary data	6.25%	Medium
Determinants of exposure to CHE: expenditure deciles (+)—use of dentistry services, inpatient service—member over 65 years (+)—employment situation of household head—education status of household head (−)—health insurance—equivalent household size (−)—gender of the head of household (female) (+)—living in the urban (−)									
31	Abolhallaje et al. (2013)	Analytical study	Article—English	2002–2005–2008	Iran	–	Secondary data	–	Medium
Determinants of exposure to CHE: employment situation of the head of household—education of the head—gender of the head of household—age of the head—number of the members of household—number of the members over 60—number of kids below 12—number of the employed persons in household—health insurance—large/small housing									
32	Davari et al. (2015)	Retrospective cross sectional study	Article—English	2004 and 2011	Chaharmahal and Bakhtiary	715 (2004) 1001(2011)	Secondary data	2004 3.4% (rural) 1.7% (urban) 2011 0% (rural) 1.7% (urban)	Medium
Determinants of exposure to CHE									
33	Homaie-Rad et al. (2017)	Before-and-after analysis	Article - English	2013 [before the reform] and 2015 [after the reform]	Guilan	1217 (2013) 1205 (2015)	Secondary data	5.75% (2013) 3.82% (2015)	Good
Determinants of exposure to CHE									
34	Homaie-Rad et al. (2016)	Cross -sectional study	Article—English	2012	Iran retirees	6307	Secondary data	0.6%	Medium
Determinants of exposure to CHE									
35	Khadivi et al. (2016)	Descriptive-analytical study	Article—Persian	2013	Construction workers in Isfahan	400	Questionnaire	4.75%	Medium
Determinants of exposure to CHE									
36	Yazdi-Feyzabadi et al. (2017)	Retrospective study	Article—Persian	2008–2014	Iranian provinces	Not reported	Secondary data	2.7%	weak
Determinants of exposure to CHE									
37	Ghafoori et al. (2014)	Descriptive–analytic study	Article—English	2012	22 districts of Tehran	784	Questionnaire	7.2%	Medium
Determinants of exposure to CHE									
38	Ahmadnezhad et al. (2019)	Cross-sectional survey	Article—English	2013–2016	Iranian households	Not report	Secondary data	3.76% (2013) 3.82% (2016)	Good
Determinants of exposure to CHE: health transformation plan									
39	Yazdi-Feyzabadi et al. (2019)	Cross-sectional survey	Article—English	2011–2017	Iranian households	Total: 76,300 38,434 (2011) 37,866 (2017)	Secondary data	1.99% (2011) 3.46% (2017)	Good
Determinants of exposure to CHE: health transformation plan had no considerable success in financial protection, requiring a review in actions to support pro-poor adaptation strategies									
40	Yazdi-Feyzabadi et al. (2018)	Descriptive study	Article—English	2008–2015	Iranian households	Total: 77,156 39,008 (2008) 38,148 (2015)	Questionnaire	2.57% (2008) 3.25% (2015)	Good
Determinants of exposure to CHE: health insurance

**Table 2:** The data extraction (patient level)

n	Author (year)	Study design	Publication type—language	Years of study	Population	Sample size	Data collection method	CHE (%)	Study quality
1	Kavosi et al. (2014)	Descriptive-analytical study	Article—English	2011	Cancer Namazi Hospital in Shiraz	245	Questionnaires	67.9%	Good
Determinants of exposure to CHE: type of insurance (relief committee–medical services) (+)—distance of the residence of the medical center—use of outpatient services—type of treatment (chemotherapy) (+)—refrained from using healthcare services (+)									
2	Moghimi et al. (2009)	Crosssectional, descriptive study	Article—Persian	2007 and 2008	Cancer-Valiasr Hospital in Zanjan	60–70	Questionnaires	52% (1386) 42% (1387)	Weak
Determinants of exposure to CHE									
3	Salehi et al. (2013)	Crosssectional (descriptive) study	Thesis—Persian	Not reported	Dialysis Patients-Hospital Dialysis Center Buali in Ardabil	200	Questionnaires	72.5%	Medium
Determinants of exposure to CHE									
4	Panahi et al. (2014)	Descriptive-analytical study	Article—Persian	2011–2012	Hospitalized patients in Tabriz	300	Questionnaires	30%	Medium
Determinants of exposure to CHE: gender of the household head (male) (−)—members over 60 years (+)—members under 12 years (+)—member with chronic illness—Non-native (+)—health insurance—access to safe water (−)—self-employed head of household (+)—education status of household head (+)—age of household head (+)- admission to a private hospital (+)—household size (+)—living in the rural (−)—wealth index (−)—marital status of household head (not married head) (−)—gender (female patients) (+)—age ) patients) (+)									
5	Anbari et al. (2014)	Cross‑sectional study	Article—English	Not reported	Markazi province	758 (total) 284 (hospitalized)	Questionnaire	11.2% (all participated) 42.6% (hospitalized)	Medium
Determinants of exposure to CHE: members aged 40–59 years old (+)—wealth index (lower levels) (+)									
6	Hajizadeh et al. (2011)	Cross‑sectional study	Article—English	2003	Inpatient services in Iran	3339	Secondary data	–	Medium
Determinants of exposure to CHE: length of stay (+)—age patients (−)—sex of the patients (male) (+)—education status of patients (−)—medical treatment insurance- social security insurance—armed forces insurance—private insurance—special organisations insurance—Imdad (relief) committee insurance- hospital owned by private sector (+)—household size (−) –wealth quintile (−)									
7	Ghiasvand et al. (2010)	Cross‑sectional study	Article—Persian	2008–2009	Hospitalized patients in 5 hospitals affiliated to Iran University of Medical Sciences	314	Questionnaire	–	Medium
Determinants of exposure to CHE: gender of the head of household (female) (+)—being native(−)—disease of family members—supplementary health insurance—household size(+)—number of hospitalizations—Household income level—housing ownership (−)									
8	Moradi et al. (2017)	Descriptive-analytical study—cross-sectional	Article—English	2015	Households with members suffering from dialysis-kidney transplant (MS)—Kurdistan province	Dialysis (87) MS (141) Kidney transplant patient (107)	Questionnaire— telephone conversations	MS (20.6%) Dialysis (13.8%) Kidney transplant patient (18.7%)	Good
Determinants of exposure to CHE: Economic status (−)—level of education (patient) (−)—supplementary insurance status (patient)—type of disease (MS)—members with special diseases in the household—living in the rural (+)—frequency of using inpatient services- use of dental care—use of rehabilitation services									
9	Almasi et al. (2016)	Analytical study—cross-sectional	Article—Persian	2014	Dialysis patients referred to Ayatollah Taleghani Hospital in Urrmia	108	Questionnaire	30%	Medium
Determinants of exposure to CHE: wealth index (−)—gender of household head (male) (−)—frequency of using dialysis services (+)—health insurance—Supplemental insurance—Members in need of care(+)—being native (−)—employment situation of the head of household									
10	Ghiasvand et al. (2014)	Cross‑sectional study	Article—English	2012	Five hospitals with tehran university of Medical Sciences	359	Questionnaire	15.05%	Good
Determinants of exposure to CHE: household Head Educational level (−)—gender of the head of household (female) (+)—hospitalization day numbers (+)—having made any out of hospital payments—quartiles’ of annual income of household (−)									
11	Juyani et al. (2016)	Cross‑sectional study	Article—English	2014	Households that at least one of their members suffers from MS—Ahvaz, Iran	322	Questionnaire	3.37%	Medium
Determinants of exposure to CHE: age of household head (−)—number of visits—gender of the household head (male) (−)—having basic health insurance coverage—household income level—house ownership (+)—household size (+)- brand of drug (foreign drugs) (+)									
12	Ghiasvand et al. (2010)	Analytical—cross-sectional study	Article—Persian	2009	Hospitalized patients in 5 hospitals affiliated to Iran University of Medical Sciences	400	Questionnaire	–	Medium
Determinants of exposure to CHE: gender of the household head (female) (+)—being native (−)—disease of family members—supplementary health insurance—household size (+)—frequency of using inpatient services—house ownership (−)—household income level (−)									
13	Rezapour et al. (2016)	Cross-sectional study	Article—English	2014	Hospitals in Hamedan	772	Questionnaire by interviews and observation	20.7%	Good
Determinants of exposure to CHE: age of household head (+)—household head educational level (−)—household size (−)—having member < 6 years (−)—having Member < 14 years (−)—having member > 60 years (+)—having own house (+)—income quintile (−)—household head employment—existence of a certain financial sources to get healthcare services (−)—disabled member in households—complementary health insurance

### CHE at population level

The rate of CHE in the studies conducted at the population level is estimated to be 4.7%, ranging from 4.1 to 5.3% at 95% Confidence Interval-CI (Table 3). The pooled estimate of CHE prevalence in Iran are shown in by the forrest plot (Fig. 3). The following results are reported with threshold level of 40% of income. The lowest percentage of CHE is reported by Homaie-Rad et al. among 6307 Iranian retirees (0.6%) [41], while the highest percentage of CHE rate is reported by Asefzadeh et al. among 100 households in Qazvin Province (24%) [26].

**Table 3:** Heterogeneity of studies

Model	Effect size and 95% interval			Test of null (2-tail)		Heterogeneity				Tau-squared			
	Point estimate	Lower limit	Upper limit	Z-value	P-value	Q-value	df (Q)	P-value	I-squared	Tau squared	Standard error	Variance	Tau
Fixed	0.041	0.057	0.040	− 1333.61	0.000	25,612.613	51	0.000	99.801	0.211	0.181	0.033	0.460
Random	0.047	0.042	0.053	− 45.900	0.000								

The studies conducted at the population level use either primary data or secondary data. A subgroup analysis was therefore performed based on the type of data used in these studies. Cochran’s Q test and $I^{2}$ index indicated a significant heterogeneity between the results of studies using primary data and those using secondary data (Table 4). The percentage of CHE reported in studies that use primary data is 11.6%, which varies between 10.4 and 13%. On the other hand, the percentage of CHE estimated in studies that use secondary data is 3%, and ranges between 2.3 and 4%.

**Table 4:** Grouping studies based on data type

Group by type of data	N	Event rate (% CHE)	Lower limit	Upper limit
Primary data	22	0.116	0.104	0.130
Secondary data	30	0.030	0.023	0.040
Overall	52	0.093	0.083	0.103

To determine the trend of CHE rates in Iran, the studies were divided into four groups based on the timeline of the studies; from 1984 to 2017. The highest percentage of CHE is observed in 2011–2017 (6.9%), while the lowest percentage of CHE is observed in 2001–2005 (4.1%) (Table 5).

**Table 5:** Group by year of studies

Group by year	N	Event rate (% CHE)	Lower limit	Upper limit
2011–2017	25	0.069	0.054	0.095
2006–2010	15	0.045	0.036	0.056
2001–2005	11	0.041	0.024	0.068
< 2001	1	0.053	0.051	0.056
Overall	52	0.053	0.050	0.055

### Factors that affect CHE at the population level

Factors that affect CHE at the population level include health insurance status; supplementary insurance status; living in rural area; age, gender, employment status and education level of the head of household; having a household member aged 60–65 years or older; number of members aged 12 years or below; number of members aged 5 years or below; having a household member with chronic illness or disabled or required care; number of working household members; marital status; and household size. The economic status of households; household expenditures; wealth index; income per capita; informal payments; expenditure per capita; and gross income by income decile groups are the economic factors reported as determinants of CHE rates (Table 6).

**Table 6:** Determinants of exposure CHE (population level)

Determinants of catastrophic health expenditures	Frequency of studies with this factor	
	Increased likelihood of CHE	Decreased likelihood of CHE
Factors related to household characteristics		
Health insurance		13
Member over 60–65 years	12	
Employment situation of household head		8
Education status of household head	2	8
Living in the urban	1	7
Member with chronic illness	4	
Supplementary insurance status of household head		3
Number of members employed in the household	1	4
Number of members under 12 years	3	
Gender of the head of household (female)	4	1
Age of household head	3	1
Disabled members	2	
Member in need of care	2	
Number of members under 5 years	1	2
Preschool children living in household		1
Marital status (married)	1	1
Household size	6	3
Household size (3 ≤ x < 6)	1	
Household size (> 7)	2	
Household economic factors		
Economic status		5
Expenditure deciles	3	
Wealth index		3
Per capita Infrastructure residential area of the household		3
Informal payment	2	
Per capita household expenditure	2	1
Income	1	
The factors related to the use of health services		
Use of Inpatient service	12	
Use of dentistry services	8	
Use of outpatient service	8	
Pharmaceutical expenses	3	
Use of medical services and diagnosis	2	
Number of use of health services	2	
Use of drug addiction cessation services	1	
Use of rehabilitation services	1	

The use of inpatient services, dental care, outpatient services, rehabilitation, drug rehabilitation, medical and diagnostic services, the frequency of receiving of healthcare services, and drug prices are other factors that affect CHE. Each of these factors can have an powerful impact on the level of CHE. Factors affecting levels of CHE must be considered and understood before allocating budgets for health. Identifying theses factors guarantee access to professionals, technologies, and necessary supplies for the promotion and recovery of their health as well as disease prevention. Health insurance status is the only variable, whose effect on facing CHE was examined in all studies. Most of the studies indicated that having health insurance reduced CHE.

### CHE at the diseases level

**Fig. 4:**
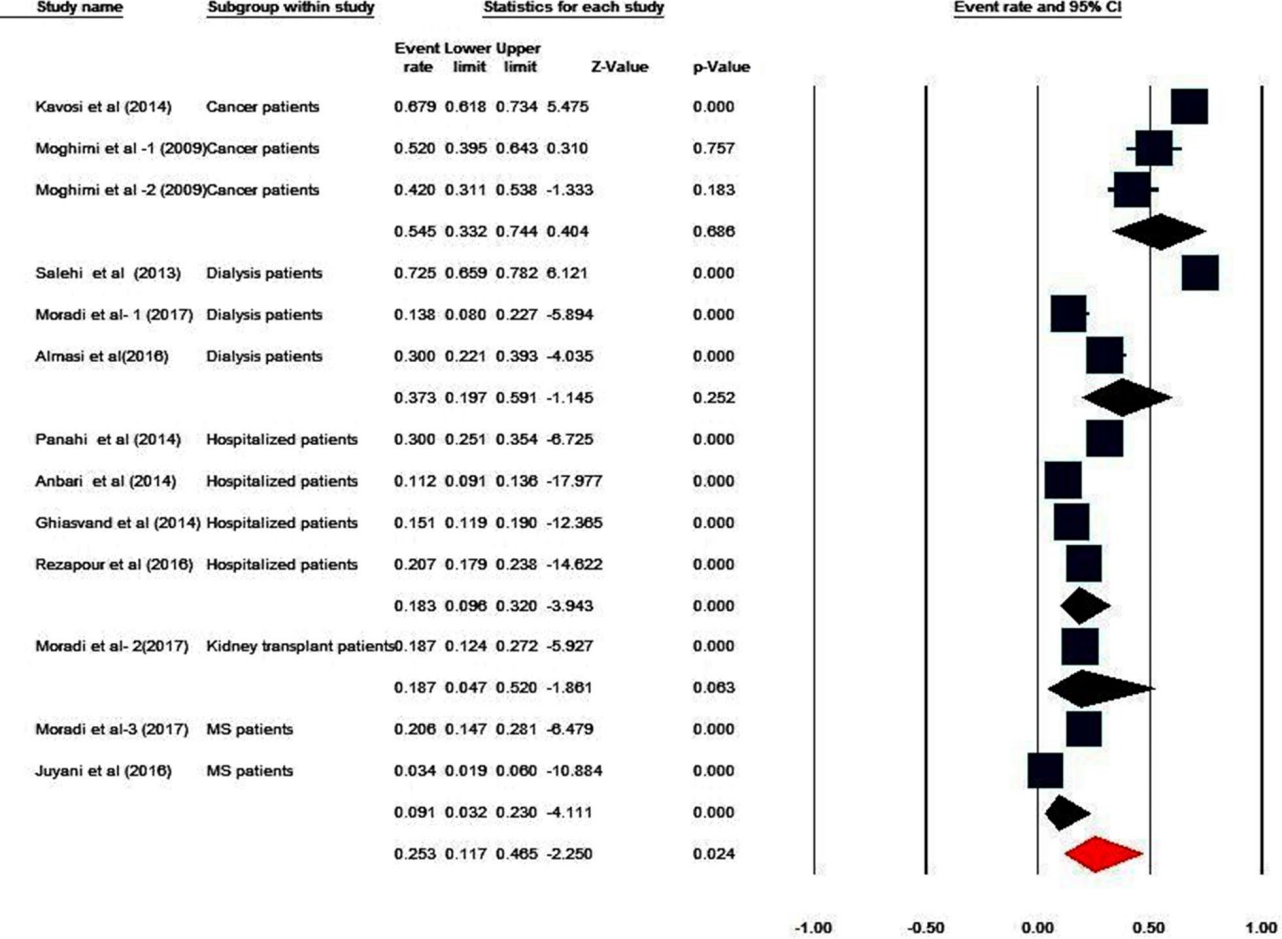
The pooled estimate of CHE prevalence obtained from subgroups’ meta-analysis (diseases level)

Due to the high heterogeneity of the studies (Q value = 544.516, df = 12, P < 0.001, I^2^ = 97.72), a random effects model was used to synthesize the results. The percentage of CHE at diseases level is 25.3%, ranging from 11.7 to 46.5% at the 95% CI (Table 7). The following results are reported with threshold level of 40% of income. The highest percentage of CHE is observed among patients undergoing dialysis (72.5%) [64], while the lowest percentage of CHE is observed among multiple sclerosis (MS) patients (3.4%) [42]. Studies were divided into groups based on disease type, and the level of CHE for each group is presented in Table 8. The highest percentage of CHE (54.5%) is observed among cancer patients (33.2–74.4% at the 95% CI) and the lowest level of CHE (9.1%) is observed among MS patients (3.2–23% at 95% CI). The pooled estimate of CHE prevalence based on the diseases level are shown in Fig. 4.

**Table 7:** Group by type of patients

Group by type of patients	N	Event rate	Lower limit	Upper limit	P-value
Cancer patients	3	0.545	0.332	0.744	0.686
Dialysis patients	3	0.373	0.197	0.591	0.252
Hospitalized patients	4	0.183	0.096	0.320	0.000
Kidney transplant patients	1	0.187	0.047	0.520	0.063
MS patients	2	0.091	0.032	0.230	0.000
Overall	13	0.253	0.117	0.465	0.024

**Table 8:** Determinants of exposure CHE (patient level)

Determinants of catastrophic health expenditures	Frequency of studies with this factor	
	Increased likelihood of CHE	Decreased likelihood of CHE
Factors related to household characteristics		
Gender of the household head (female)	6	
Supplementary insurance status (patient)		5
Health insurance		4
Non-native	4	
Members over 60 years	2	
Employment situation of the head of household		2
Disease of family members	2	
Members with special diseases in the household	1	
Member with chronic illness	1	
Members under 12 years	1	
Type of insurance (relief committee–medical services)	1	
Distance of the residence of the medical center	1	
Disabled member in household	1	
Members in need of care	1	
Education status of patients		2
Education status of household head	1	2
Self-employed head of household	1	
Household size	4	2
Access to safe water		1
Age of household head	2	1
Having member < 6 years		1
Having member < 14 years		1
Marital status of household head (not married head)		1
Sex of the patients (male)	1	1
Age (patients)	1	1
Members aged 40–59 years old	1	
Living in the rural	1	1
Household economic factors		
Household income level		5
Wealth index		4
Housing ownership	2	2
Economic status		1
Having made any out of hospital payments	1	
Existence of a certain financial sources to get healthcare services		1
The factors related to the use of health services		
Frequency of using inpatient services	3	
Hospitalization day numbers	2	
Admission to a private hospital	2	
Use of outpatient services	1	
Frequency of using outpatient services	1	
Use of rehabilitation services	1	
Brand of drug (foreign drugs)	1	
Refrained from using healthcare services	1	
Use of dental care	1	
Type of treatment (chemotherapy)	1	
Frequency of using dialysis services	1	
Frequency of using inpatient services	3

### Factors affecting CHE at the disease level

Factors affecting CHE rate at the disease level were categorized into three groups: (a) socio-demographic factors, (b) economic factors and (c) disease-related factors. Socio-demographic factors included: gender of the head of household, basic insurance status and insurance type, supplementary insurance status, being native, having a household member older than 60 years old, employment status of the head of household, having a household members with illness, having members with special diseases, having members with chronic diseases, having members aged 12 years or below, having members that are disabled or require care, education level of the patient, education level of the head of the household, household size, age of the head of household, having a member aged 6 years or below, having a member aged 14 years old or below, marital status of the head of household, age and gender of the patient, having a member aged 40–59 years old, access to clean water, distance between the place of residence and health centers, and living in a rural areas. Economic factors included: income, wealth index, property ownership, economic status, OOPs, and having specific resources for paying healthcare costs. Disease-related factors included: frequency of using inpatient services, hospitalization days, admission to private hospitals, frequency of using outpatient services, use of rehabilitation services and dental care, drug brands, avoiding healthcare services due to financial problems, type of treatment in cancer patients (e.g. chemotherapy), and dialysis frequency.

## Discussion

The overall percentage of CHE in Iran is estimated to be 4.7% based on the synthesis of the reviewed studies. Further analysis reveals that the percentage of CHE is 11.6% in studies that use primary data and 3% in studies that use secondary data. Studies with primary data use the WHO survey and interviews for data collection, while those with secondary data use data from the Household Income and Expenditure Survey (HIES) which is collected regularly by the Iran Statistics Center (ISC). The 8.6% difference is likely due to differences in sample size and the instruments used to collect data. Evidence shows that questionnaires that are designed based on the WHO survey more accurately measure the health expenditures of households compared with HIES survey, since the former is specifically designed to measure health expenditures [39, 65, 66]. A systematic review conducted by Ghorbanian et al. in 2015 revealed that studies that use the WHO survey for data collection report higher levels of CHE than studies that use the HIES survey. Their review estimates levels of CHE at 3.91% at the population level [39], which is lower than the value estimated in this paper. A likely reason for this inconsistency is the higher number of studies that use primary data included in this study compared with the Ghorbanian et al. review. In another study of levels of CHE across Iran’s provinces over a 7-year period (2008–2014), the highest percentage of CHE (5.2%) is observed in Fars Province and the lowest percentage of CHE (0.7%) is observed in South Khorasan Province [60].

In this review, the identified studies were divided into four groups based on the timeline of the studies (1984–2015). The results show that the number of studies on CHE has increased during this period, reaching its highest level between 2011 and 2015. Moreover, it is revealed that the level of CHE increased from 2001 to 2015, with the highest percentage of CHE observed between 2011 and 2015. Despite the policies developed and actions taken to reduce OOPs, levels of CHE are still high and have reached their highest levels in recent years. This is mainly caused by the increasing costs of healthcare, which includes the cost of medications and use of complex treatments that require specialized facilities and equipment. This creates financial difficulties for households and puts pressure on the strained health budgets of different countries [67]. Another reason for rising CHE rates is the financing mechanisms used in various health systems. In under-developed and low-income countries, OOPs consistute a substantial proportion of health financing and adequate prepayment mechanisms are often lacking [15].

At the level of diseases, the percentage of CHE is estimated to be 25.3%. The highest level of CHE is observed among cancer patients (54.5%) and the lowest among MS patients (9.1%). In a study by Kavoosi and colleagues on CHE in a southern Iranian city, CHE rate is reported to be 67.9% among cancer patients [12]. Other studies have shown that households with cancer patients have the highest levels of catastrophic health spending [12, 68]. It is therefore critical to review the existing financing policies regarding these patients and to develop fair health financing strategies for these vulnerable groups in Iran.

Cancer patients in other countries are facing catastrophic health spending as well due to the high costs of treatment. A 2014 study in India reports 53% of patients with non-communicable diseases are exposed to CHE, with cancer patients experiencing the highest percentage of CHE (74%) [69]. In another study, which was conducted in 2017 in Malaysia on colorectal cancer patients, the authors find that 47.8% of patients’ families experience CHE [27]. In addition, a study across eight Southeast Asian countries reports that 31% of cancer patients experience financial catastrophe [70]. In South Korea, Lee and colleagues show that CHE in the households without disabled members was 27.6%, 13.2%, 7.8%, and 5.1% with the threshold at 10%, 20%, 30%, and 40% respectively. Factors associated with incidence of CHE included the number of household members, household income, receiving public assistance, having a member over 65 years and household head’s employment status [71]. A study by Ma and colleagues finds that the incidence of catastrophic expenditure in China experienced a 0.70-fold change between 2010 (12.57%) and 2016 (8.94%). One of the most important factors affecting CHE is household income [72]. In Kimani’s study in Kenya, among those who utilize health care, 11.7% experience CHE and 4% are impoverished by health care payments [73].

Among the social factors that affect levels of CHE at the population level, health insurance status (reported in 13 studies) and employment of the head of household (reported in 8 studies) are the most important factors that reduce levels of CHE. Having a member aged 60–65 years or older in the household (reported in 12 studies) is the most important factor that increases levels of CHE. Households that have no health insurance coverage or use services that are not covered in an insurance plan have to spend a higher portion of their income and possibly sell assets to purchase health services. Risk pooling and proper prepayment mechanisms provided by insurance companies can therefore play a significant role in protecting people against CHE and ensure their access to healthcare [15, 74–78]. However, a study conducted in China shows that health insurance coverage can increase levels of CHE, since people with health insurance can be encouraged to use more health services [79]. Employment status of the household head is another major factor that affects levels of CHE and can reduce the likelihood of experiencing financial hardship by increasing the financial capacity of the household [13, 80]. Older individuals are more susceptible to various diseases and are more in need of healthcare. Having older individuals in the household therefore increases its health expenditures and, consequently, increases its chance of experiencing CHE [8]. In a number of other studies conducted in different countries, the presence of an older individual has been shown to increase the risk of incurring CHE [7, 78, 81–85].

Among economic factors, the economic status and wealth index of households are the most important factors in decreasing levels of CHE, while high household expenditure is the most important factor in increasing levels of CHE. Better economic status and higher wealth index indicate that the household has more resources and a higher payment capacity; thus, higher wealth index is associated with lower risk of incurring CHE [17, 63]. Other studies conducted in India [80], Mexico [82], Turkey [7], Vietnam [85], and Burkina Faso [86] have also reported the economic status of households as a key determinant of CHE. In disease-related factors, the frequency of using inpatient services, outpatient services, and dental care are the most important factors affecting levels of CHE. This is in line with the findings from studies conducted in other settings, which indicate that the risk of incurring CHE increases with the frequency of using inpatient [86–88] and outpatient care [84].

At the disease level, the gender of the head of household, basic insurance status, supplementary insurance status, and being native are four major social determinants of CHE. Female heads of households have less job opportunities and a lower chance of employment, and they are mostly supported by their children or relatives, charities, and retirement pensions. As a result, female headed households are more likely to incur CHE [76, 84]. The farther the distance from the place of residence to health centers, the higher the direct non-medical costs of the households (e.g. transportation and accommodation costs) [13]. Non-native households are therefore more likely to incur CHE [12, 75]. Similarly to the population level, income and wealth index (reported in 5 and 4 studies respectively) are the most important economic factors that reduce the likelihood of patients’ households incurring CHE. Among disease-related factors, the frequency of using inpatient services, hospitalization days, admission to private hospitals, and the frequency of using outpatient services are the most important factors and are positively associated with the likelihood of patients’ households being exposed to CHE [12, 36, 38, 40, 42, 52]. Studies in different settings have shown that increased usage of healthcare services is associated with a higher risk of incurring CHE [86].

## Conclusions and recommendations

The present review provides a comprehensive picture of fairness in Iran’s health system in terms of addressing CHE. The results demonstrate the high percentage of households exposed to CHE in Iran. This rate is significantly higher in vulnerable groups and in households with certain diseases. Fore some diseases, studies show that more than half of patients incur CHE. Therefore, it is critical to review existing health financing policies and to develop new policies to protect people against financial hardship. Designing a health financing system that protects demographics and diseases with greater exposure to CHE can contribute to health equity and significantly reduce levels of CHE.

Countries can reduce involved in illness by relying more on prepayment and less on OOPs. In that way, people contribute to funding health services in a predictable fashion, and are not required to suddenly find money to pay for services when they fall ill unexpectedly. Catastrophic expenditures do not automatically disappear with rising income. National health financing systems must be designed not only to allow people to access services when they are needed, but also to protect households from financial catastrophe, by reducing out-of-pocket spending. In the long term, the aim should be to develop mandatory prepayment mechanisms, such as social health insurance, tax-based financing, or some mix of prepayment mechanisms. In moving towards such a system, flexible short-term responses will be needed, which will depend on the stage of economic development of the country and on the social and political context. Policy-makers will need to consider how to expand population coverage through prepayment mechanisms; protect the poor and disadvantaged; design a benefits package; and decide the level of cost sharing by the patients.

## Supplementary information

**Additional file 1.** Search strategy.

## Data Availability

No additional data available.
