# Supplementary material for: Determinants of catastrophic health expenditures in Iran: a systematic review and meta-analysis
Source: Cost Eff Resour Alloc. 2020 May 15;18:17. doi: 10.1186/s12962-020-00212-0 (PMC7229629; doi:10.1186/s12962-020-00212-0)
Supplement: Supplementary file 1 — Additional file 1. Search strategy. [file 12962_2020_212_MOESM1_ESM.docx]

Thanks a lot for correction and proof reading. We corrected and checked proof, please see pdf file that we performed changes and identified in pdf file.
